# The Effects of Visual Cues, Blindfolding, Synesthetic Experience, and Musical Training on Pure-Tone Frequency Discrimination

**DOI:** 10.3390/bs9010002

**Published:** 2018-12-24

**Authors:** Cho Kwan Tse, Calvin Kai-Ching Yu

**Affiliations:** Department of Counselling & Psychology, Hong Kong Shue Yan University, Hong Kong 999077, China; kcyu@hksyu.edu

**Keywords:** frequency difference limens, blindfold, visual cues, auditory-visual synesthesia, gliding frequencies, perceptual limit, common resource theory, multiple resource model

## Abstract

How perceptual limits can be reduced has long been examined by psychologists. This study investigated whether visual cues, blindfolding, visual-auditory synesthetic experience, and musical training could facilitate a smaller frequency difference limen (FDL) in a gliding frequency discrimination test. Ninety university students, with no visual or auditory impairment, were recruited for this one-between (blindfolded/visual cues) and one-within (control/experimental session) designed study. Their FDLs were tested by an alternative forced-choice task (gliding upwards/gliding downwards/no change) and two questionnaires (Vividness of Mental Imagery Questionnaire and Projector–Associator Test) were used to assess their tendency to synesthesia. The participants provided with visual cues and with musical training showed a significantly smaller FDL; on the other hand, being blindfolded or having a synesthetic experience before could not significantly reduce the FDL. However, no pattern was found between the perception of the gliding upwards and gliding downwards frequencies. Overall, the current study suggests that the inter-sensory perception can be enhanced through the training and facilitation of visual–auditory interaction under the multiple resource model. Future studies are recommended in order to verify the effects of music practice on auditory percepts, and the different mechanisms between perceiving gliding upwards and downwards frequencies.

## 1. Introduction

Humans can listen to and detect a variety of sounds under different conditions. To be specific, a normal human behavioral frequency difference limen (FDL), which is the just noticeable difference in hearing, is 1.22% to 4.02% in 140 Hz, and 0.25% to 2.50% between the frequency of 80–400 Hz [1,2]. However, the FDL measured by the electrical frequency-following response emanating directly from the brainstem neurons is even smaller (75%) than the behavioral FDL [1]. Accordingly, FDL might vary from person to person, and the brainstem can detect a smaller FDL than the behavioral perception. It would be interesting to investigate the factors for these discrepancies, and to find methods to improve the behavioral perception. To calculate FDL, Nelson, Stanton, and Freyman proposed a square-root function between log (FDL) and frequency [3], whereas Micheyl, Xiao, and Oxenham defined the equation in one of their models, as follows [4]:

$$E\left[d_{i}\left(f,d,s\right)\right]=\beta_{f}{\left(\frac{f}{1000}\right)}^{\gamma^{f}}+\beta_{d}{\left(\frac{d}{100}\right)}^{\gamma^{d}}+\beta_{s}{\left(\frac{s}{10}\right)}^{\gamma^{s}}+r_{i}+\alpha$$

Regarding this, the stimulus presentation frequency (*f*), duration (*d*), and level (*s*) would affect the just-noticeable difference in pure-tones [4]. In particular, a greater decrease of FDL was found in a smaller tonal duration (*d*; 5 ms) and a larger sensation level (*s*; 80 dB SL), in which the effect remained unchanged with a tonal duration of 200 ms; and was more significant in low frequency (*f*; 200 Hz) [5,6]. The gliding frequencies were also found to have a higher detection rate of frequency change, as compared with the discrete frequencies [7]. These factors are related to the presentation of frequencies and might decrease FDL. Yet, by managing these controllable factors, there was still a discrepancy between the behavioral FDL and the FDL recorded from the frequency-following responses in the brainstem neurons [1]. Therefore, this study attempted to examine other factors, such as the visual–auditory interaction, synesthetic experience, and musical training, so as to minimize the FDL of the gliding frequencies, which is related to interactions with the visual system and attention allocation. Many researchers have conducted studies on FDL [8,9,10,11], but most of them examined discrete frequency discrimination, which required participants to discriminate two separate pure-tones. There have been very few studies about the FDL of gliding frequencies [12,13,14] and the features of the neural systems that are sensitive to detecting gliding frequencies (frequency shift detectors—FSDs) [13]. The present study can fill in the literature gap and shed light on the understanding of gliding frequency perception, as well as approaches to overcome the perceptual limit in auditory perception. For example, it would be beneficial for training for the ability to recognize each pitch without an external reference—namely the absolute pitch [15].

Studies have found that visual–auditory interactions are beneficial for increasing the accuracy of perception [16]. Hirata and Kelly found a bigger improvement in phoneme learning when English speakers were allowed to look at the lip movement during Japanese audio training [17]. In contrast, the improvement was very little when the English speakers were provided with the hand gesture or listened to the audio tape alone. As demonstrated in this example, combining the visual and auditory information during the perception process can achieve a more accurate perception. Considering the attention models that explain the visual–auditory interaction, Wicken proposed a multiple resource model, in which different modalities were processed in different channels, which would facilitate each other while activities in the same modality would compete each other and therefore hinder the performance [18]. Based on the rule of cross-modal similarities, Marks studied four inter-sensory relations, which were pitch-lightness, pitch-brightness, loudness-brightness, and pitch-object form [19]. He demonstrated that the congruent presentation of auditory and visual stimuli led to a higher accuracy and faster response than the incongruent presentation. For example, he showed that during a discrete frequency discrimination task, pairing a higher frequency with a brighter visual stimulus resulted in a shorter reaction time than pairing with a dim visual stimulus. This phenomenon illustrated that when there is a similarity in the visual and auditory information, a faster and more accurate response can be obtained [19,20]. Consistent with this early study, there is event-related potential (ERP) and functional magnetic resonance imaging (fMRI) evidence that auditory cortical responses could be enhanced when the tones are paired up to an attended visual stimulus [21]. As demonstrated by the studies above, the congruent presentation of visual stimuli and auditory stimuli would result in a faster reaction time and higher response accuracy through the cross-modal interaction. The studies above revealed that different sensory modalities are interlinked and can facilitate responses. Therefore, visual cues can be a potential factor to facilitate auditory perception, in which a congruent visual cue might facilitate a better auditory perception. Therefore, this study applied the cross-modal interaction to test the minimization of FDL, through the interaction with visual modality. The cross-modal interaction is similar to the Stroop task (i.e., naming a color word that is printed in another color), in which the task performance is hindered by a mismatched condition between the meaning of the word and its color, while it is facilitated by a matched condition between the meaning of the word and its color [22]. The current study targets the improvement of auditory perception with the presence of visual cues. As a congruent visual cue could predict a more accurate response, it is expected that it could help to reduce the behavioral FDL.

Despite the benefits from the cross-modal interaction, the visual–auditory interaction, on certain occasions, can distort perception. Take the sound-induced flash illusion as an example; the perception of two auditory tones being paired with one flash light would be perceived as two instances of flash lights [23]. Similarly, the ventriloquist effect was defined as the perception of voice that seems coming from a direction other than the true place [24]. Moreover, the Mcgurk effect is an example of merged visual–auditory information; that is, the auditory perception of “ba” and “ga” to be “da” is a form of intermediate perception [25]. In other words, this intermediate perception does not improve either the visual or auditory perception, but generates other percepts. As demonstrated by the experiments above, vision could sometimes distort the auditory perception. In contrast to the multiple resource theory that has been mentioned in the previous paragraph [18], Kahneman proposed a single pool of attention resources (i.e., common resource theory [26]), and early research indicated the shared capacity in processing visual and auditory discrimination by demonstrating the difficulties of discriminating pitch and light intensity at the same time [27]. Recent research also found that visual tasks that demanded low attentional resources could improve auditory thresholds [28]. Moreover, the evidence that visual and auditory processing occurred at the central level rather than the two peripheral mechanisms seemed to support the common central resources model [29]. A recent study also suggested sharing visual and auditory attentional resources, in that visual–spatial and auditory–spatial information did not facilitate performance [30]. It revealed that resources are limited, and vision and auditory processing are two dependent processing mechanisms that compete for the central resources. Vision and auditory processing would influence each other. For example, visual processing was impaired with concurrent spoken messages [31]; the duration for the perception of effort and exertion in physical exercise was longer in the people who used visual and auditory senses together [32]. Furthermore, the cross-modal Stroop task demonstrated that the hearing of auditory color words could distract and slow down people’s performance in a color naming task [33]. In view of these studies, attention was shown to be limited and shared by different processing. Thus, to improve auditory processing, the common resource model would suggest allocating more attentional resources to auditory perception than visual perception, through visual deprivation. As resources are limited, it might be that humans would allocate more resources to auditory processing when vision is deprived. Studies have revealed that blindness can bring a range of improvements in auditory perception [34,35]. Similarly, a study also showed that being blindfolded for ninety-minutes enhanced the performance of harmonicity perception. This could be explained by the metamodal model, in which the deprivation of visual inputs would rapidly release nonvisual inputs (i.e., auditory and tactile) from suppression, because the domination of visual sensory in the striate cortex is halted [36]. In an animal study, Petrus et al. found an improvement in the frequency selectivity and discrimination of the primary auditory cortex (A1) neurons after visual deprivation for 6–8 days [37]. Another animal study also revealed that visual deprivation would refine the intra- and inter-laminar connections in the auditory cortex (A1) [38]. In connection with this, Williams demonstrated that vision is responsible for two-thirds of the brain’s electrical activity when opening eyes [39]. In view of this high consumption of resources in visual perception, blindfolding would suppress visual processing and allocate more attentional resources to auditory processing. From the review above, the common central resource model suggests that blindfolding would enhance auditory perception by channeling more resources to auditory processing. Therefore, blindfolding is another potential factor that may minimize FDL.

Synesthesia is an involuntary sensory experience where the stimulation of one modality evokes the sensation of another modality [40,41]. In general, around 5% of the population have experienced one type of synesthesia [42], such as auditory–tactile synesthesia, chromesthesia (sound-to-color synesthesia), grapheme-color synesthesia, and auditory–visual synesthesia. There are two hypotheses regarding the mechanisms of synesthesia. The cross-activation hypothesis suggests that the synesthetic experience is due to the excessive neural connections between the adjacent cortical areas [43]. In contrast, the disinhibited-feedback hypothesis proposes that synesthesia is the consequence of the inhibition failure between the brain areas [44]. No matter which mechanism is correct, the two sensory modalities are inter-linked, and a cross-modal interaction plays a part in synesthesia. This points to the possibility of improving one’s perception through the synesthetic experience from another modality. Indeed, there is evidence that the synesthetes have a better visual ability. By measuring the vividness of the mental image through the Vividness of Mental Imagery Questionnaire (VVIQ), the synesthetes shared a major characteristic of having a more vivid mental image than the non-synesthetes [45]. Despite the fact that most of the subjects in the study were linguistic-color synesthetes, a few were colored music and visual-taste synesthetes. Furthermore, a study demonstrated that the participants with a high VVIQ score could be trained to acquire the grapheme-color synesthesia through associative learning, which involved extensive memory and reading exercises [46]. Although auditory perception was not involved in this study, it revealed that a synesthetic experience could be induced in non-synesthetes. It also suggested that non-synesthetes with a high VVIQ score could be trained to become “synesthete” and acquire the advantages of a cross-modal interaction in auditory perception. Synesthetic experiences involving one modality might favor a better performance in another modality through a stronger association between the modalities. A neurological study found an increased activation in the left inferior parietal cortex (IPC) of the auditory–visual synesthetes when compared to the non-synesthetes [47]. As IPC is responsible for multimodal integration and feature binding, the researchers believed that the auditory–visual synesthetes had a more enhanced sensory integration ability than the non-synesthetes. Accordingly, a synesthetic experience might facilitate a better visual–auditory interaction and thus improve auditory perception. In addition, in the case of a visual flash causing auditory synesthetic experiences, the synesthetes demonstrated an excellent ability in a difficult visual task involving rhythmic temporal patterns [48]. In this case, the advantage of performing the visual task was not only owing to the visual system, but also because of the “hearing” of the rhythmic temporal patterns in the auditory perception. Therefore, when two senses interacted and intertwined together, they benefited each other. It is worth examining whether a visual synesthetic experience would facilitate or impair the frequency discrimination. With reference to the cross-modal interaction, it could enhance the auditory discrimination ability and result in a smaller FDL. The study of visual synesthetic experience would provide pragmatic information about the inter-sensory processing and clarify the argument between cross-modal perception and unimodal perception in synesthesia.

Musical training has been found to be a crucial factor for improving auditory perception. Musicians have a half FDL, earlier pitch change detection, and better ability to discriminate frequencies, in comparison with non-musicians [49,50]. A previous experiment demonstrated that the threshold of pitch discrimination for musician participants was six times smaller than that for non-musician participants [51]. It further indicated that the non-musician participants needed at least 14 h of training to attain the similar pitch-discrimination threshold as the musician participants [51]. Therefore, ordinary people can acquire an enhanced pitch-discrimination ability if they receive musical training. As indicated by a larger amplitude of N2b and P3 responses during attentive listening, professional musicians also showed a faster and more accurate pitch detection than non-musicians [52]. Furthermore, musicians seemed to have a different neuroanatomy from non-musicians, such as an increased amount of grey matter, and therefore their neural encoding of sound is superior [53]. Therefore, musical training might somehow “train’ the brain to acquire better auditory abilities. Besides visual–auditory interaction, musical training can be another important factor for improving the hearing experience. The present study examined whether musical training would expand the limit in auditory perception. Despite the previous evidence that musicians have a smaller FDL, this study examined the effect of musical training on FDL in gliding frequency perception. Although Gottfried and Riester already demonstrated that music students have a better performance in pitch glide identification tests, their results were based on accuracy, not FDL [14]. Thus, the present study could provide information of the FDL of gliding frequencies.

The present study aimed to investigate the effects of visual cues, blindfolding, synesthetic experience, and musical training on behavioral FDLs. Both multiple resource and common resource models imply an advantage of these four factors for frequency discrimination. Therefore, it was hypothesized that either providing visual cues or minimizing visual inputs could reduce FDL. Moreover, given the stronger communication between the visual and auditory modalities in synesthesia, a smaller FDL was expected to be found in participants with a synesthetic experience. Finally, it was hypothesized that the participants with musical training would have a smaller FDL.

## 2. Materials and Methods

### 2.1. Participants

Ninety university students (37 males and 53 females) were recruited to participate in this study, aged 17 to 25 years (*M* = 20.32 years old, standard deviation (SD) = 1.30). All of the participants were asked to complete the informed consent and a self-report background questionnaire before the experiment, in order to ensure normal hearing and visual ability.

Nine participants’ data were screened out because of program errors (i.e., the frequency range was not reduced after a correct answer or was reduced after an incorrect answer).

### 2.2. Materials

The experimental setup was adapted from the study of Demany, Carlyon, and Semal (2009) [54]. The experiment was conducted in a quiet environment. All of the tested frequencies were sinusoidal waveform pure-tones that were generated through MATLAB and delivered through Earpods. An Asus UX430U laptop with the Realtek High Definition Audio and WDM audio device was used in this experiment. The reference frequencies were 110, 440, and 1760 Hz, with a sound pressure level (SPL) of 65 dB. These three reference frequencies were chosen because they represent the second octave, fourth octave (middle octave), and the sixth octave in the piano [55], which constitute the common frequency range in a music piece. Specifically, 440 Hz was chosen because it was the tuning preference in orchestra [56].

All of the initial target frequencies were set a semitone higher or lower than the reference frequency, as it was the basic detectable difference of a frequency in music. In this study, FDL was defined by the difference between the reference frequency and the initial target frequency. Table 1 shows all of the reference frequencies and the initial target frequencies in this study.

**Figure 1:**
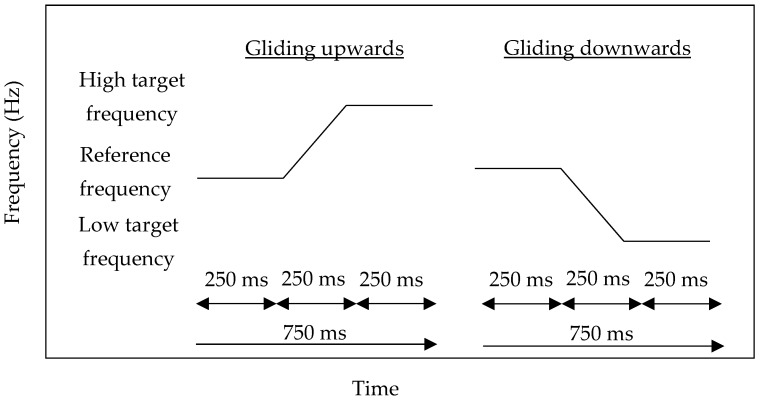
Schematic representation of the stimulus presentation of the gliding upwards (**left**) and gliding downwards (**right**) pure-tones.

In each condition, as gliding frequencies have a higher detection rate of frequency change than discrete frequencies [12], the two pure-tone frequencies were arranged to glide upwards or downwards, smoothly and randomly in 750 ms, instead of being separated by a silent interval. Within 750 ms, the first 250 ms was the reference frequency, the second 250 ms was the gliding effect, and the last 250 ms was the target frequency. An unlimited pause of the inter-stimulus interval was given in each trial for responding. The schematic representation of the stimuli is shown in Figure 1.

Interventions and questionnaires were applied in order to investigate the effects of the four factors on FDL. In particular, the effects of visual cues and blindfolding were examined by interventions, while the effects of synesthesia and music experience were examined through questionnaires.

**Figure 2:**
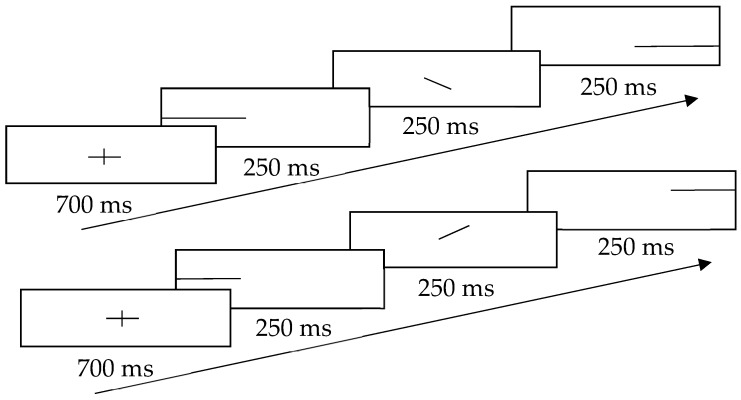
Schematic representation of the visual cues with the upper part showing gliding downwards and the lower part showing gliding upwards.

The design of visual cues was adapted from the study by Ben-Artzi and Marks (1995), which revealed a positive relationship between the pitch change and the position of the dot [20]. To minimize the efforts of the searching dots, this study adopted a continuous straight line as the visual cue. This line showed the gliding direction when a participant listened to the sound track and identified the change of the frequency. The schematic representation of visual cues is shown in Figure 2. To prevent participants from noticing the answers directly from the cues, both congruent and incongruent cues were presented to them. For the congruent cue, the line went up or down in the middle of the screen, according to the gliding direction in the sound track; for the incongruent cue, the line went in the opposite direction to the gliding direction in the sound track. To prevent habituation and to indicate the start of the next trial, a fixation cross (+) was flashed at the beginning of each trial.

Participants in the blindfolded group were instructed to complete the frequency discrimination test with an eye mask.

To assess the synesthetic experiences, this study adopted the VVIQ and the Projector–Associator Test (PAT) from the Synesthesia Battery on https://www.synesthete.org/. This battery was a standardized test to investigate and study synesthesia [40].

The VVIQ scale consists of 32 five-point Likert-scale questions, which evaluate the vividness of the mental imagery. The Cronbach’s alpha for the VVIQ scale was 0.92 in this study.

The PAT consists of 12 five-point Likert-like items, which measure the types of synesthetic experiences. The Cronbach’s alpha for the PAT was 0.88. Those of the projector scale and the associator scale were 0.78 and 0.83, respectively.

In addition to VVIQ and PAT, the background information of the participants, the presence of a perfect/absolute pitch, the years of musical training, and the presence of visual–auditory synesthetic experience were collected.

### 2.3. Procedures

Before the experiment started, a frequency discrimination test (Lutman, 2004) was applied to ensure the ability to discriminate frequencies [57]. This test contained 14 trials and required participants to choose a higher tone between the reference frequency (500 Hz) and the target frequency (seven trials with a 5% change, or seven trials with a 2% change). Participants needed to obtain seven accurate responses or more to pass the test. In this study, all of the participants passed this screening test (*M* = 91.14% of accuracy, SD = 1.45).

After that, five practice trials were given for demonstrating the operation and to ensure that all of the participants were confident with the experimental procedures. All of the participants were required to perform both an experimental session and a control session; they were randomly assigned to participate in one of the sessions first. For the experimental session, the participants were randomly divided into two conditions—visual cues and blindfolding. In the visual cues experimental session, a total of 180 trials were presented to each participant. They comprised fifteen trials of gliding upwards and fifteen trials of gliding downwards in three frequency levels (low/middle/high) and two types of visual cues (congruent/incongruent). All of the trials were randomly presented.

In the blindfolded experimental session, a total of 90 trials were presented to each participant. It was composed of fifteen trials of gliding upwards and fifteen trials of gliding downwards in three frequency levels (low/middle/high). All of the trials were randomly presented, and the participants were required to put on an eye mask during the whole experimental session.

After finishing the experimental session, the VVIQ and PAT questionnaires were distributed. 

Next, the control session was given with a similar procedure to the blindfolded condition, except that the participants were instructed to focus on the fixation cross (+) on the screen. To eliminate the habituation effect, the fixation cross was flashed once before every trial. Ninety trials were randomly presented, and were different from those of the blindfolded experimental session. 

In each trial, participants were told to discriminate whether there was an upward change, downward change, or no change in the frequency tone. To indicate their response, they were instructed to press an upward arrow (↑), downward arrow (↓), or right arrow (→) in the keyboard when they thought the operating sound track was increasing in frequency, decreasing, or remained unchanged, respectively. For each correct answer, the frequency change, which was the difference between the reference frequency and the target frequency, decreased by half. On the other hand, each incorrect response increased the change by one-fourth of the original change.

## 3. Results

Extreme outliers of FDL in each variable were screened out before the analysis. They were defined as Q_1_ − 3 * interquartile range (IQR) or Q_3_ + 3 * IQR. As the distribution of the FDL values was not normal, and the auditory perception and, in particular, perceiving an octaves match closely with logarithmic scales, the collected FDLs (in Hz) were transformed into a logarithmic scale as follows [58]:

FDL (in octaves) = log_2_ (f_2_/f_1_).

### 3.1. Visual Cues and FDL

Six Wilcoxon signed-rank tests were conducted to investigate the effect of visual cues on FDL compared with the control session (looking at a fixation cross).

**Figure 3:**
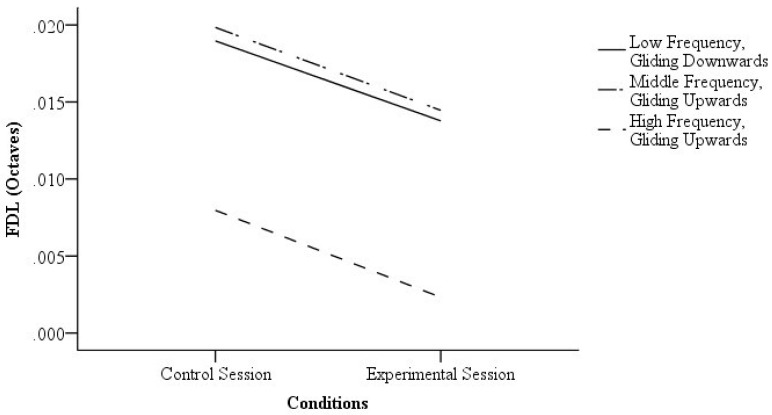
The frequency difference limens (FDLs) of the control session and experimental session of the significant results.

Three tests showed a significant difference between the experimental session and control session. At first, for the “low frequency, gliding downwards” condition, the FDL was significantly lower when the participants were provided with visual cues (*Z* = −3.10, *p* = 0.00, *r* = 0.50) with a large effect size. For the “middle frequency, gliding upwards” condition, the FDL was also significantly lower when the participants were provided with visual cues (*Z* = −2.15, *p* = 0.03, *r* = 0.36) with a medium effect size. Furthermore, for the “high frequency, gliding upwards” condition, the FDL was significantly lower when the participants were provided with visual cues (*Z* = −4.10, *p* = 0.00, *r* = 0.72) with a large effect size. The FDLs of the control session and experimental session are shown in Figure 3.

The experimental session and control session showed no significant difference in the other three conditions (*p*-values of the tests > 0.05). Table 2 shows the details of the statistical results.

### 3.2. Blindfolding and FDL

Six Wilcoxon signed-rank tests were conducted to investigate the effect of blindfolding on FDL. However, no significant difference was found between the experimental session and the control session (*p*-values of the tests > 0.05). Table 3 presents the statistical results with the *p*-values and effect sizes.

### 3.3. Experience of Visual Synesthesia in Auditory Perception

In total, eight participants reported the presence of visual associations when hearing music. The mean score of the VVIQ was 3.28 (SD = 0.64), while that of the PA was −0.22 (SD = 0.67). Twelve Mann–Whitney *U*-tests were conducted to evaluate the hypothesis that people with a synesthetic experience would have a smaller FDL. No significant differences were found (*p*-values > 0.05). Table 4 summarizes the statistical results and the descriptive statistics.

In the experimental session of “middle frequency, gliding downwards”, control session of “middle frequency, gliding upwards”, and both the control and experimental sessions of “low frequency, gliding upwards” and “high frequency, gliding downwards”, the median FDL of the participants who had a synesthetic experience was smaller than that of the participants who did not have a synesthetic experience.

Correlations of the VVIQ score, PAT score, and FDLs were conducted to further examine the relationship between the visual synesthetic experience and auditory discrimination ability. Nonetheless, only a significant negative relationship was found between the FDLs in the control session of “high frequency, gliding downwards” and the VVIQ score (*r*_s_ = −0.34; *p* = 0.00). There was no significant relationship between the VVIQ score and FDLs. There was also no significant correlation between the PAT scores and FDLs (*p*-values > 0.0).

### 3.4. Musical Training

As the perfect pitch/absolute pitch was a confounding variable in this study, twelve Mann–Whitney *U*-tests were conducted to evaluate the difference of the FDLs between the perfect pitch/absolute pitch participants (*n* = 5) and the remaining participants. Only under the condition of “middle frequency, gliding downwards, control session” did the perfect pitch/absolute pitch participants show a lower FDL (*Mdn* = 0.003 octaves) than the other participants (*Mdn* = 0.019 octaves; *U* = 69; *p* = 0.02; *r* = 0.28), which showed a small effect size. Yet, as the other conditions did not show a significant difference in FDL (*p*-values > 0.05), the results suggested no difference between the perfect pitch/absolute pitch participants and the normal participant in the present study.

Fifty participants had been engaged in musical training (*M* of training years = 6.19; SD = 4.80), while 31 participants reported to have no musical training. The presence of musical training was defined as having received vocal training or played a musical instrument, such as piano, violin, flute, or guitar. Twelve Mann–Whitney *U*-tests were conducted to examine the effect of musical training experience on the FDLs. Six tests showed significant differences (*p*-values < 0.05). Table 5 presents the statistical results with the *p*-values, medians, ranges, and effect sizes.

In general, the participants with musical training showed a significantly smaller FDL than the participants without musical training in the conditions of “low frequency, gliding upwards”, “middle frequency, gliding downwards, experimental session”, “middle frequency, gliding upwards, experimental session”, and “high frequency, gliding downwards”.

Another two independent sample *t*-tests were conducted to investigate the effect of musical training experience on the VVIQ score and PAT score. Apart from these, the participants with musical training (*M* = −3.34; SD = 0.78) also showed a more negative PAT score than the participants without musical training (*M* = −0.02; SD = 0.54; *t*(79) = −2.13; *p* = 0.04; *d* = 0.50) with a medium effect size. Nonetheless, the participants with musical training (*M* = 3.36; SD = 0.68) did not show a significantly higher VVIQ score than the participants without musical training (*M* = 3.14; SD = 0.54; *t*(79) = 1.50; *p* = 0.14; *d* = 0.35) with a small effect size.

## 4. Discussion

### 4.1. Visual Cues and FDL

The present study found that providing visual cues might be an effective way to reduce FDLs. In the conditions of “low frequency, gliding downwards”, “middle frequency, gliding upwards”, and “high frequency, gliding upwards”, the experimental session showed a significantly smaller FDL than the control session. This finding partially supports the hypothesis.

According to the multiple resource theory, facilitation occurs when different tasks are processed in different channels, and interference arises when different tasks are processed in the same modality [18]. On the basis of the current results, the visual modality and auditory modality are under separate attention control [59], such that the visual modality might not compete the resources with the auditory modality. Hence, the congruent visual cues could facilitate the frequency discrimination task in serval conditions.

The neural systems that are sensitive to detect gliding frequencies (frequency shift detectors—FSDs) have an optimal range of 120 cents (0.10 octaves) [13]. However, the findings of this study suggest that the frequency shift detectors are also sensitive to changes that are smaller than 120 cents in some conditions. In particular, the visual–auditory interaction might reduce the perceptual limit in some cases.

However, there was no significant difference between the control session and the experimental session in the conditions of “low frequency, gliding upwards”, “middle frequency, gliding downwards”, and “high frequency, gliding downwards”. As the FDL applied in this study was continuously being reduced to a level smaller than 120 cents, the findings suggest that in some conditions, the FDLs beyond this optimal range are not susceptible to modifications.

In some conditions, either the gliding downwards or gliding upwards frequencies condition was found to have a significant difference between the control session and the experimental session. This might imply two different mechanisms in perceiving gliding downwards and gliding upwards frequencies. Likewise, it might also suggest that the FSDs respond differently to the gliding downwards and gliding upwards frequencies. Although Luo et al. (2007) found that humans are equally sensitive to the upward and downward frequency-modulated tone sweeps [60], the FDLs of the gliding downwards frequencies were found to be lower than that of the upwards frequencies [61].

### 4.2. Blindfolding and FDL

According to the common resource theory [26], as cognitive resources are limited, blindfolding was assumed to minimize the FDL by allocating more resources to the auditory perception. Yet, in the present study, temporary blindfolding failed to result in this effect. This might be due to the insufficient time for acquiring the analogous activity of brain plasticity. According to Lewald (2007), the initiation of auditory–visual cross-modal plasticity requires at least ninety minutes of visual deprivation before having an enhancement effect in sound localization [62]. Similarly, in animal studies, the sensory enhancement due to visual deprivation required six to eight days [37,38]. Therefore, temporary blindfolding might not be effective for instantly reducing the FDL, and blindfolding only during the experimental session was not enough to induce a plasticity and to improve the hearing experience in the present study.

### 4.3. Synesthetic Experience and FDL

Participants who had a synesthetic experience did not have a significantly lower FDL in comparison with the participants without a synesthetic experience. This indicated that cross-modal interaction in synesthesia fails to improve the ability to discriminate frequencies.

In fact, there is evidence that the sensory enhancement is related to the same modality of the synesthetic experience, such as color sensitivity being enhanced only in those synesthetes who have a color synesthetic experience [63]. To enhance the auditory perception, sounds should be the synesthetic experience instead of the stimuli that trigger the synesthetic experience. Therefore, the visual synesthetic experiences examined in the present study might not be conducive to auditory perception. 

### 4.4. Musical Training and FDL

Musical training is an important factor for auditory perception, given the finding that the presence of musical training minimized the FDL in most of the conditions. In fact, the benefits of musical training on enhancing auditory perception have already been documented in the literature [49,50,51]. 

Bianchi, Santurette, Wendt, and Dau explained that musical training can enhance pitch representation in the auditory system, and thus improve auditory perception [64]. Likewise, Schellenberg and Moreno asserted that musically trained participants would have a more comprehensive mental representation and more profound memory, because musical training involves the cross-modal encoding of stimuli [65].

In this connection, the advancement of frequency discrimination might also be related to a better association between the different modalities. In the current study, as indicated by their lower PAT scores, the participants who had musical training were strong associators. An associator tends to have synesthesia experiences in the mind’s eye rather than in the external space [40,66]. 

It is reasonable that musicians have a stronger association between sensory modalities, as learning musical instruments involves the interaction of several modalities and high-order cognitive functions [67]. For example, the crowding effect is a phenomenon where each musical note is crowded by an adjacent note on the five-lined staff, which slows down the pace of music-reading. However, with extensive practice, musicians would acquire specific music-reading visual skills to tackle this crowding effect, and eventually develop better visual spatial resolution [68]. As illustrated by this example, musical training not only enhance auditory perception, but also visual spatial ability. 

### 4.5. Limitations and Future Studies

There was no observed pattern comparing the results of the gliding upwards and gliding downwards frequencies. Although the underlying reason was still unclear, perceiving gliding downwards and gliding upwards frequencies seem to be governed by two dissimilar mechanisms. Future studies are suggested in order to verify these frequency-detection mechanisms.

One of the major limitations of this study was the fatigue effect. As the study proceeded around thirty minutes and consisted of both experimental and control sessions, the participants might feel tired when participating in the experiments. Considering this, they might react to the program randomly. Hence, the recorded FDL might not reflect the true FDL of the participants, which would impair the internal validity of the FDL. In the future, applying another more simple experimental design is recommended to find the FDL. To be specific, an “ABX” paradigm, in which participants are asked to answer whether tone X is equal to tone A or tone B, is a simpler method to determine the FDL. 

Another limitation was the design of the visual cues. Participants might ignore the visual cues and might only focus on the sound track. Therefore, the visual cues might not be effective for inducing the expected visual–auditory interaction. To improve the study, a pilot test is recommended. For example, before testing the FDL, it is better to apply visual cues to a discrete frequency discrimination task with a larger and more obvious frequency change. By checking the accuracy, the effectiveness of the visual cues can be guaranteed.

The VVIQ score and the background questionnaire were used to identify the synesthetic experiences. Very few participants in the current study reported such experiences. It is suggested that the sample size should be enlarged so as to recruit participants who are potentially synesthetic.

Future studies may also be carried out to explore the mechanisms of perceiving gliding upwards and downwards frequencies, given the two different results observed for the FDL in the gliding upwards and downwards frequencies.

Last but not least, the effect of musical training was found to be prominent in minimizing FDLs. Future studies should explore the effect of musical practices on other perceptual limits, such as visual acuity and picture resolution.

## 5. Conclusions

In conclusion, this study investigated the effects of visual cues, blindfolding, synesthetic experience, and musical training on behavioral FDLs. The multiple resources model was partially supported, as visual cues could reduce FDLs in some conditions. In contrast, FDLs were not significantly lowered by the blindfolding and synesthetic experience. The participants who had a synesthetic experience and those who did not performed similarly in the FDL tasks. However, very few participants in the current study reported synesthetic experiences. Therefore, whether synesthetes might have a similar FDL requires further investigation. Finally, musical training could lower FDLs. These results suggest that the perceptual limits in auditory perception can be reduced through the practice and facilitation of visual–auditory interaction.

## Figures and Tables

**Table 1:** The reference frequencies and initial target frequencies from the first, fourth, and seventh octave.

Reference Frequency (Hz)	High Initial Target Frequency (Hz)	Low Initial Target Frequency (Hz)
110	116.54	103.83
440	466.16	415.3
1760	1864.66	1661.22

**Table 2:** Statistical analysis of the Wilcoxon signed-rank tests of the effect of visual cues on the frequency difference limen (FDL).

FDL		*N*	*Mdn* (Octaves)	*Range* (Octaves)	*Z*	*p*	*r*
Low frequency, gliding downwards	Control session	39	0.019	0.12	−3.10	0.00	0.50
	Experimental session		0.014	0.09			
Low frequency, gliding upwards	Control session	32	0.034	0.28	−0.84	0.40	0.15
	Experimental session		0.014	0.48			
Middle frequency, gliding downwards	Control session	35	0.019	0.05	−0.69	0.49	0.12
	Experimental session		0.014	0.09			
Middle frequency, gliding upwards	Control session	35	0.020	0.05	−2.15	0.03	0.36
	Experimental session		0.014	0.04			
High frequency, gliding downwards	Control session	32	0.017	0.05	−0.36	0.72	0.06
	Experimental session		0.014	0.03			
High frequency, gliding upwards	Control session	32	0.006	0.02	−4.10	0.00	0.72
	Experimental session		0.002	0.01			

**Table 3:** Statistical analysis of the Wilcoxon signed-rank tests of the effect of blindfolding on FDL.

FDL		*N*	*Mdn* (Octaves)	*Range* (Octaves)	*Z*	*p*	*r*
Low frequency, gliding downwards	Control session	42	0.019	0.12	−1.53	0.13	0.24
	Experimental session		0.019	0.12			
Low frequency, gliding upwards	Control session	39	0.020	0.28	−0.20	0.84	0.03
	Experimental session		0.020	0.28			
Middle frequency, gliding downwards	Control session	38	0.019	0.05	−0.02	0.99	0.00
	Experimental session		0.019	0.05			
Middle frequency, gliding upwards	Control session	38	0.008	0.05	−0.42	0.68	0.07
	Experimental session		0.008	0.05			
High frequency, gliding downwards	Control session	33	0.008	0.05	−0.64	0.53	0.11
	Experimental session		0.008	0.05			
High frequency, gliding upwards	Control session	40	0.008	0.02	−0.11	0.91	0.02
	Experimental session		0.006	0.02			

**Table 4:** Statistical analysis of the Mann–Whitney *U*-tests of the presence/absence visual association.

FDL		Visual Association	*N*	*Mdn* (Octaves)	*Range* (Octaves)	*Z*	*U*	*p*	*r*
Low frequency, gliding downwards	Control session	Presence	8	0.033	0.12	−0.83	240.50	0.41	0.09
		Absence	73	0.019	0.12				
	Experimental session	Presence	8	0.016	0.09	−0.33	271.00	0.74	0.04
		Absence	73	0.014	0.12				
Low frequency, gliding upwards	Control session	Presence	8	0.020	0.28	−1.39	191.00	0.17	0.16
		Absence	68	0.049	0.28				
	Experimental session	Presence	7	0.014	0.09	−1.13	168.50	0.26	0.13
		Absence	65	0.020	0.48				
Middle frequency, gliding downwards	Control session	Presence	7	0.019	0.01	−0.46	211.00	0.64	0.05
		Absence	67	0.019	0.05				
	Experimental session	Presence	7	0.011	0.03	−0.65	241.00	0.52	0.07
		Absence	70	0.014	0.09				
Middle frequency, gliding upwards	Control session	Presence	7	0.008	0.02	−1.01	188.00	0.31	0.12
		Absence	69	0.020	0.05				
	Experimental session	Presence	7	0.008	0.03	−0.14	234.00	0.89	0.02
		Absence	69	0.008	0.05				
High frequency, gliding downwards	Control session	Presence	7	0.008	0.05	−0.71	186.00	0.48	0.08
		Absence	63	0.019	0.05				
	Experimental session	Presence	8	0.007	0.09	−1.50	162.50	0.13	0.18
		Absence	60	0.014	0.09				
High frequency, gliding upwards	Control session	Presence	7	0.008	0.02	−0.83	202.50	0.41	0.09
		Absence	71	0.008	0.02				
	Experimental session	Presence	7	0.003	0.01	−0.16	226.00	0.87	0.02
		Absence	67	0.003	0.02				

**Table 5:** The statistical analysis results of the Mann–Whitney *U*-tests of the musical training.

FDL		Musical Training	*N*		*Mdn* (Octaves)	*Range* (Octaves)	*Z*	*U*	*p*	*r*
Low frequency, gliding downwards	Control session	Yes		50	0.019	0.12	−1.19	655.00	0.24	0.13
		No		31	0.019	0.12				
	Experimental session	Yes		50	0.019	0.09	−1.35	637.00	0.18	0.15
		No		31	0.005	0.12				
Low frequency, gliding upwards	Control session	Yes		48	0.020	0.28	−3.21	377.50	0.00	0.37
		No		28	0.049	0.28				
	Experimental session	Yes		47	0.014	0.48	−3.44	298.00	0.00	0.41
		No		25	0.049	0.48				
Middle frequency, gliding downwards	Control session	Yes		47	0.019	0.04	−0.17	620.00	0.86	0.02
		No		27	0.019	0.05				
	Experimental session	Yes		49	0.014	0.09	−2.07	512.00	0.04	0.23
		No		29	0.019	0.09				
Middle frequency, gliding upwards	Control session	Yes		49	0.008	0.05	−1.73	510.00	0.08	0.20
		No		27	0.020	0.05				
	Experimental session	Yes		49	0.008	0.05	−2.93	394.00	0.00	0.33
		No		29	0.014	0.05				
High frequency, gliding downwards	Control session	Yes		47	0.008	0.05	−2.55	347.00	0.01	0.30
		No		23	0.019	0.04				
	Experimental session	Yes		46	0.014	0.09	−2.87	290.00	0.00	0.35
		No		22	0.019	0.09				
High frequency, gliding upwards	Control session	Yes		48	0.008	0.02	−0.24	697.00	0.81	0.03
		No		30	0.008	0.02				
	Experimental session	Yes		47	0.003	0.01	−0.84	560.00	0.40	0.10
		No		27	0.003	0.02				
